# FEASIBILITY AND ACCEPTABILITY OF INSPIRATORY MUSCLE STRENGTH TRAINING AMONG OLDER ADULTS IN MYSORE, INDIA

**DOI:** 10.1093/geroni/igad104.3352

**Published:** 2023-12-21

**Authors:** Raghavi karnam, Nagalambika Ningaiah, Shivamma Nanjaiah Nanjaiah, Nagaveni Bhyra, Kiranmayee Muralidhar, Purnima Madhivanan, Karl Krupp

**Affiliations:** Public Health Research Institute of India, Mysore, Mysore, Karnataka, India; Public Health Research Institute of India, Mysore, Mysore, Karnataka, India; Public Health Research Institute of India, Mysore, Mysore, Karnataka, India; Public Health Research Institute of India, Mysore, Mysore, Karnataka, India; Public Health Research Institute of India, Mysore, Mysore, Karnataka, India; University of Arizona, Tucson, Arizona, United States; Mezcoph, Tucson, Arizona, United States

## Abstract

Hypertension, a major risk for cardiovascular and cerebrovascular diseases, affects 1.3 billion globally and 229 million in India. While exercise has shown to lower blood pressure, 40% of older Indians report inadequate physical activity. Inspiratory Muscle Strength Training (IMST), a novel breathing exercise, shows promise for controlling hypertension, but little is known about its acceptability among older Indian adults. This study examined feasibility and acceptability of IMST in older adults with hypertension in Mysore, India. Between November-2022 and January-2023, 31 non-smoking adults aged 65 - 80 years, with a blood pressure □140/90 mm Hg were enrolled in a 6-week interventional study after informed-consent. Participants used a Power-breathe Plus breath trainer for 30 breaths a day, 5 days a week, for 6-weeks, weekly increasing resistance to a maximum of 30% of their Maximum Inspiratory Pressure. Participants underwent medical assessment, interviewer-administered survey to collect data on demographics, depression, cognition, physical performance, pulmonary function and acceptability, at baseline and post-intervention. Univariate analysis was conducted. Of 31 participants, 20% were male and 55% were on antihypertensive. Four participants discontinued by week two. Post-intervention, 22% had □10 mm Hg decrease in systolic- pressure and 37% □10 mm Hg decrease in diastolic- pressure. About 59% showed improvement in depression screening scores, 40% had improved cognitive scores and 48% reported improved physical-performance scores; 80% reported IMST was useful and 90% reported device was user-friendly; 45% reported improvements in breathlessness and exercise-tolerance. IMST was acceptable, feasible and safe as a hypertension intervention for older adults in Mysore, India.

